# A dose titration protocol for once‐daily insulin glargine 300 U/mL for the treatment of diabetes mellitus in dogs

**DOI:** 10.1111/jvim.17106

**Published:** 2024-06-03

**Authors:** Antonio Maria Tardo, Linda Mary Fleeman, Federico Fracassi, Alisa Saule Berg, Aria L. Guarino, Chen Gilor

**Affiliations:** ^1^ Department of Veterinary Medical Sciences University of Bologna Bologna Italy; ^2^ Animal Diabetes Australia Collingwood Victoria Australia; ^3^ Department of Small Animal Clinical Sciences College of Veterinary Medicine, University of Florida Gainesville Florida USA; ^4^ BluePearl Pet Hospital Rockville Maryland USA

**Keywords:** basal‐bolus insulin therapy, continuous glucose monitoring, diabetic dogs, FreeStyle Libre, Toujeo

## Abstract

**Background:**

In purpose‐bred dogs, insulin glargine 300 U/mL (IGla300) has long duration of action, peakless time‐action profile, and low potency, making it suitable for use as a basal insulin.

**Hypothesis:**

To evaluate IGla300 in client‐owned diabetic dogs monitored using a flash glucose monitoring system (FGMS).

**Animals:**

Ninety‐five client‐owned diabetic dogs, newly diagnosed or previously treated with other insulin formulations, with or without concurrent diseases.

**Methods:**

Prospective multi‐institutional study. Clinical signs and standardized assessment of FGMS data, using treatment and monitoring guidelines established a priori, guided dose adjustments and categorization into levels of glycemic control.

**Results:**

The initial IGla300 dose was 0.5 U/Kg q24h for newly diagnosed dogs and (median dose [range]) 0.8 U/Kg (0.2‐2.5) q24h for all dogs. Glycemic control was classified as good or excellent in 87/95 (92%) dogs. The IGla300 was administered q24h (1.9 U/kg [0.2‐5.2]) and q12h (1.9 U/kg/day [0.6‐5.0]) in 56/95 (59%) and 39/95 (41%) dogs, respectively. Meal‐time bolus injections were added in 5 dogs (0.5 U/kg/injection [0.3‐1.0]). Clinical hypoglycemia occurred in 6/95 (6%) dogs. Dogs without concurrent diseases were more likely to receive IGla300 q24h than dogs with concurrent diseases (72% vs 50%, respectively; *P* = .04).

**Conclusions and Clinical Importance:**

Insulin glargine 300 U/mL can be considered a suitable therapeutic option for once‐daily administration in diabetic dogs. Clinicians should be aware of the low potency and wide dose range of IGla300. In some dogs, twice‐daily administration with or without meal‐time bolus injections may be necessary to achieve glycemic control. Monitoring with FGMS is essential for dose titration of IGla300.

**Abbreviations**
BCS: body condition score
BGCs: blood glucose curves
DM: diabetes mellitus
FGMS: flash glucose monitoring system
IG: Interstitial glucose concentration
IGla100: insulin glargine 100 U/mL
IGla300: insulin glargine 300 U/mL
NPH: neutral protamine Hagedorn
PZI: protamine zinc insulin

## 1 INTRODUCTION

Insulin treatment is the cornerstone of diabetes mellitus (DM) management in dogs. Ideally, insulin treatment in dogs should mimic the physiology of endogenous insulin secretion, which is characterized by a “basal‐bolus” pattern.^1^ However, to minimize costs and the need for multiple daily injections, insulin treatment in dogs traditionally has relied on the use of intermediate‐acting insulin suspensions administered at the time of feeding.^2^ These formulations however are associated with some drawbacks such as the need to match insulin administration to consistent feeding, marked day‐to‐day variability, and increased risk of hypoglycemia.^3^, ^4^, ^5^, ^6^, ^7^ Diabetology in humans has shifted to using recombinant insulin analogs which are designed to closely mimic physiologic insulin secretion and to have minimal within‐day and between‐day variability, which are important features in minimizing hypoglycemic events.^8^, ^9^

Insulin glargine is a recombinant human insulin analog in which asparagine at position A21 is replaced with glycine and 2 arginine residues are added to position B30.^8^ This synthetic molecule is soluble at a pH of 4 (as supplied) but at physiologic pH (in the SC tissues) forms microprecipitates, slowing its absorption after injection.^10^ Insulin glargine 300 U/mL (IGla300; Toujeo, Sanofi‐Aventis, Bridgewater, New Jersey) is biochemically identical to insulin glargine 100 U/mL (IGla100) but is 3 times more concentrated,^11^ which results in lower potency, longer duration, and a flatter time‐action profile compared with IGla100.^12^ Several studies in people have shown that IGla300 is superior to IGla100 in maintaining glycemic control while decreasing day‐to‐day variability and frequency of hypoglycemia.^11^, ^13^, ^14^, ^15^, ^16^ In dogs, IGla300 was shown to have long duration of action, a relatively peakless time‐action profile, and low potency.^17^ In dogs with toxin‐induced DM, IGla300 administered twice daily, showed lower day‐to‐day variability compared with lente insulin.^7^ These properties make IGla300 a good candidate for use as basal insulin in dogs, and clinical trials evaluating it in client‐owned diabetic dogs are warranted.

The Freestyle Libre flash glucose monitoring system (FGMS) has revolutionized the management of DM in dogs.^18^, ^19^, ^20^, ^21^, ^22^, ^23^, ^24^, ^25^ The device measures interstitial glucose concentrations (IG) on a minute‐by‐minute basis for up to 14 days, via a disc‐shaped sensor with a small catheter inserted under the skin. The FGMS does not require calibration, is accurate, and well tolerated by dogs.^18^ Recent studies have demonstrated that the FGMS allows more accurate identification of glucose nadirs, postprandial hyperglycemia, hypoglycemic episodes, and day‐to‐day variations in glycemic control compared with serial blood glucose curves (BGCs).^20^, ^21^, ^22^, ^23^, ^24^ These advantages might be particularly evident when monitoring dogs treated with basal insulin such as IGla300.

Our study was designed to evaluate the feasibility of a treatment protocol using IGla300 as basal once‐daily insulin in client‐owned diabetic dogs with FGMS monitoring used for dose titration.

## 2 MATERIALS AND METHODS

Our study was designed as a prospective multi‐institutional data collection study using guidelines established a priori for a novel insulin treatment and dose titration protocol. Data were collected from 3 referral centers (Animal Diabetes Australia, Victoria, Australia; Department of Small Animal Clinical Sciences of the University of Florida, Gainesville, Florida, USA; Veterinary Teaching Hospital of the University of Bologna, Bologna, Italy) from January 2021 to January 2023. The timeframe of the study was not predetermined, and we chose to stop case inclusion when the number of cases was sufficient to meet the objectives of our study. Data were generated from routine clinical cases in which IGla300 was used to treat DM and FGMS monitoring was used for dose titration. Attending clinicians at the 3 institutions were asked to enter data into a shared spreadsheet on Google Drive. Before patient recruitment, 3 of the authors (LMF, FF, CG). developed detailed treatment and monitoring guidelines for IGla300 dose titration based on clinical experience and previously published data.^7^, ^17^ These a priori guidelines were followed by all attending clinicians throughout the study period under the supervision of 1 of the authors (LMF, FF, CG) at each institution. However, throughout the study period and for all patients included in the study, treatment, and monitoring decisions were at the discretion of the attending clinician with complete disregard to the contemporaneous data collection. The trial was approved by the Scientific Ethics Committee of the University of Bologna (protocol number 101123/2023) and by the University of Florida Institutional Animal Care and Use Committee (protocol number 202300000146). This research received no external funding.

### 2.1 Diabetes diagnosis and inclusion criteria

Diagnosis of DM was performed according to the Agreeing Language In Veterinary Endocrinology (ALIVE) criteria established by the European Society of Veterinary Endocrinology (ESVE).^26^ Client‐owned dogs, both newly diagnosed or previously treated with other insulin formulations, were included. Dogs having already received insulin were transitioned to IGla300 if their DM was poorly controlled or if owners expressed desire to minimize the number of daily injections (ie, using a long‐acting insulin formulation once‐daily). Dogs treated with corticosteroids or progestagens and dogs with concurrent acute (eg, diabetic ketoacidosis [DKA]) and chronic (eg, Cushing's syndrome) disorders also were included. Patients with DKA or other concurrent acute illnesses affecting overall health and life expectancy (eg, acute pancreatitis) were included after clinical signs of the concurrent disorder resolved and the dogs' general condition had improved. Diagnostic investigations for concurrent diseases were performed at the discretion of the attending clinician. There were no dietary restrictions and, in most cases, diet was unchanged during the transition to IGla300. However, the feeding method frequently was changed from a strict 12‐hourly protocol of feeding at the times of insulin injections to more frequent feeding of smaller meals or to a different twice daily feeding schedule.

Dogs only were included if their caretakers were able to perform home monitoring using the FGMS. The FGMS sensors were applied in the hospital or at home by owners. Owner perception of clinical signs and standardized assessment of FGMS data using the treatment and monitoring guidelines that were established a priori informed dose adjustments and final categorization into level of glycemic control. For glycemic control categorization, a 4‐point scoring system was employed, synthesizing owners' perception of clinical signs into actionable categories: (a) poor/insufficient control: moderate to severe clinical signs, requiring a change in treatment; (b) moderate control: mild to moderate clinical signs, a change in treatment might be required (if undesired weight loss was a component or glycemic control was deemed insufficient for the individual dog based on clinical signs); (c) good control: mild clinical signs, and no change in treatment required; and (d) excellent control: no clinical signs, and no change in treatment required. Interstitial glucose concentration (IG) was continuously monitored using the FGMS until achievement of IG between 70 and 250 mg/dL (4‐14 mmol/L) for >50% of the time, averaged over a 7‐day period, or until glycemic control was deemed appropriate for the individual case based on clinical signs. The number of days required for dose titration was measured from the start of treatment with IGla300 to the establishment of 1 of those 2 outcomes. There was no specified maximum time for the dogs to achieve those outcomes, and dogs that remained uncontrolled throughout the study period were classified as having poor/insufficient glycemic control. Clinical hypoglycemia was defined as IG <60 mg/dL (3.3 mmol/L) associated with the presence of clinical signs (eg, weakness, tremor, ataxia, collapse, seizures).

### 2.2 A priori guidelines for dose titration of IGla300

#### 2.2.1 Initial dose

For newly diagnosed diabetic dogs, the recommended initial IGla300 dose was 0.5 U/kg, administered SC q24h. The dose was rounded down to the nearest whole unit, and based on estimated ideal body weight rather than actual body weight in thin (body condition score [BCS] <4/9) or overweight (BCS ≥6/9) dogs. The dogs' owners chose their preferred time for administering the injection.

For dogs previously treated with another insulin formulation (with the exception of insulin detemir) administered q12h, the q24h starting dose for IGla300 was calculated by adding 33% to the previous q12h insulin dose, rounded down to the nearest whole unit. For example, if the dog was previously treated with 15 U q12h, the recommended starting dose of IGla300 was 20 U q24h. The first dose of IGla300 was administered 12 hours after the last dose of the previous insulin. For dogs that were already treated with another insulin formulation administered q24h, the initial IGla300 dose was the same as the dose of the previous insulin, administered at the same time of the day.

For dogs previously treated with insulin detemir, the initial q24h dose of IGla300 was calculated either by first multiplying the current q12h dose by 4 before adding 33%, or by multiplying the current q24h dose by 4.^27^

#### 2.2.2 Rapid dose titration based on FGMS data

A dose increase of 10% to 30% (or by 1 U for dogs weighing <8 kg) was recommended every 1 to 3 days as long as the IG nadir was >350 mg/dL (>19 mmol/L). When the IG nadir was <350 mg/dL (<19 mmol/L), before making additional dose adjustments, it was recommended to monitor for 3 to 5 days or until a consistent daily pattern emerged. The guidelines in Table 1 were recommended for dose adjustments based on the observed daily pattern during this period.

**TABLE 1:** Guidelines for insulin glargine 300 U/mL (IGla300) dose‐adjustments based on flash glucose monitoring system (FGMS) data during induction of therapy.

IG nadir and/or mean	Graphic illustration of interstitial glucose pattern	Recommended dose adjustments for	
		Dogs >8 kg	Dogs <8 kg
Nadir 150‐350 mg/dL (8.3‐19 mmol/L)		↑10%‐30% q24h	↑1 U q24h
Nadir 80‐150 mg/dL (4.4‐8.3 mmol/L), or nadir <80 mg/dL (<4.4 mmol/L) and mean IG >120 mg/dL (>6.6 mmol/L)	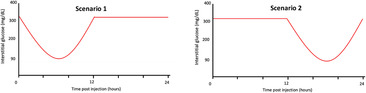	Switch to q12h dosing (with a 30% dose reduction per injection) and re‐evaluate the following 3‐5 days. Adjust Toujeo dose to achieve nadir between 90 and 300 mg/dL (5‐17 mmol/L)	
	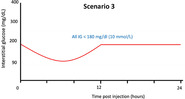	No change	
	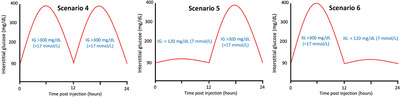	Maintain IGla300 q24h and add meal‐time bolus injections Scenario 4: at hours 0 and 12 Scenario 5: at hour 12 Scenario 6: at hour 0 OR changing the timing and/or quantities fed at meals	
Nadir <80 mg/dL (<4.4 mmol/L) and mean IG <120 mg/dL (<6.6 mmol/L)		↓10%‐30% q24h	↓1 U q24h

Abbreviations: IG, interstitial glucose concentrations.

#### 2.2.3 Indications for change to q12h dosing with or without addition of a bolus insulin injection

An increase in dosing frequency of IGla300 was recommended (from q24h to q12h) when the IG nadir was 80 to 150 mg/dL (4.4‐8.3 mmol/L), or nadir <80 mg/dL (<4.4 mmol/L) and mean IG >120 mg/dL (>6.7 mmol/L), and a period of approximtately 12 hours during each 24 hour cycle when IG results were all >300 mg/dL (>17 mmol/L). The period of high IG could occur at any part of the 24 hour cycle (eg, 0‐12 hour or 12‐24 hour post‐injection; Table 1: scenario 1 or 2). When switching from q24h to q12h dosing, it was recommended to decrease the IGla300 dose by approximately 30% per injection (ie, the total daily dose was increased by 40%), and to first administer the new dose 24 hour after the last q24h injection. After the change in frequency, the dosing decisions were to be made based on the same criteria as for q24h dosing (see previous section, “Rapid dose titration based on FGMS data”), including 3 to 5 days of monitoring and establishing a new pattern before re‐evaluating the dose.

In diabetic dogs receiving IGla300 q24h or q12h, in which a consistent pattern of postprandial hyperglycemia emerged and control of clinical signs was not achieved, it was recommended to add a meal‐time bolus injection to manage postprandial hyperglycemia. This procedure was indicated if a substantial period of IG >300 mg/dL (>17 mmol/L) consistently occurred for 4 to 8 hours after ≥1 meals during each 24 hour period (Table 1: scenario 4, 5, or 6). In these cases, Neutral Protamine Hagedorn (NPH) insulin (either as NPH or as a 70/30 NPH/regular insulin mix) or porcine lente insulin was added at ≥1 meal times at a starting dose of 0.25 U/kg, rounded down to the nearest whole unit, and based on estimated ideal body weight rather than actual body weight in thin (BCS <4/9) or overweight (BCS ≥6/9) dogs.

### 2.3 Flash glucose monitoring system

The IG measurements were acquired with a validated FGMS (FreeStyle Libre, Abbott Laboratories Ltd, Chicago, Illinois).^18^ Sensor placement was performed as previously described.^18^ More than 1 generation of Freestyle Libre (ie, Freestyle Libre 1 and Freestyle Libre 2) was used during the study period.

### 2.4 Statistical analysis

Statistical analysis was performed using commercial statistical software packages (GraphPad Prism 7, San Diego, California). Descriptive statistics were generated to characterize the study population. Continuous variables were presented as median and range (minimum and maximum value). Categorical variables were described with frequencies, proportions, or percentages. Differences between dogs with or without concurrent diseases for categorical (ie, frequency of insulin administration) and numerical variables (ie, level of glycemic control, total insulin dose, and days to achieving glycemic control) were analyzed using the Fisher's exact test and the Mann‐Whitney test, respectively. Dogs treated with topical or systemic medications that may affect glycemic control (eg, corticosteroids, progestagens) were included in the “concurrent diseases” group. Dogs with incomplete data were excluded from the analysis. The level of significance was set at *P* < .05.

## 3 RESULTS

### 3.1 Study population

One‐hundred and six client‐owned dogs were enrolled in the study. Among these, 11 were excluded because of incomplete data (n = 5), adverse events unrelated to the treatment of DM (n = 5; including acute illness [n = 3], ophthalmic surgery complications [n = 2]), and loss to follow‐up (n = 1). Of the remaining 95 dogs, 14/95 (15%) were newly diagnosed with DM, and 81/95 (85%) were previously treated with other insulin formulations. Pre‐study insulins included porcine lente (40/81, 49%), NPH (20/81, 25%), IGla100 (6/81, 7%), IGla100 as basal and NPH as bolus (12/81, 15%), IGla100 as basal and porcine lente as bolus (2/81, 2%), and regular insulin (1/81, 1%). The median age was 10 years (1.5‐16.1 years). There were 43 spayed females, 51 neutered males, and 1 intact male. At the time of enrollment, median body weight was 8.3 kg (1.2‐35.8), and median BCS was 5/9 (1.5‐8/9). Forty‐two different breeds were represented. The most common breeds included mixed breed (18), Pomeranian (7), Miniature Schnauzer (7), and Miniature Poodle (6). During the study period, the type of diet remained unchanged in 83 dogs and was modified, as deemed necessary by the managing clinician, in 12 dogs.

### 3.2 IGla300 treatment

The median (range) IGla300 starting dose was 0.8 (0.2‐2.5) U/kg q24h. At the end of the study, 56/95 (59%) dogs were receiving IGla300 q24h (median dose, 1.9 U/kg [range, 0.2‐5.2]), and 39/95 (41%) dogs IGla300 q12h (1.9 U/kg/day [0.6‐5.0]). Considering all dogs, the final insulin dose was 1.6 (0.2‐5.2) U/kg per injection and the total daily insulin dose was 1.9 (0.2‐5.2) U/kg/day. Meal‐time bolus injections (30/70 regular/NPH insulin [n = 3], porcine lente insulin [n = 2]) were added in 5/95 (5%) dogs (3 dogs receiving IGla300 q24h and 2 dogs IGla300 q12h). Bolus insulin was administered q24h in 3 dogs and q12h in 2 dogs. The median bolus insulin dose was 0.5 (0.3‐1.0) U/kg per injection.

### 3.3 Clinical outcomes

Glycemic control was classified as excellent in 62/95 (65%) dogs, good in 25/95 (26%) dogs, moderate in 7/95 (7%) dogs, and poor/insufficient in only 1 dog. The median time to achieve glycemic control was 16 (3‐99) days. The time to achieve glycemic control was ≤30 days in 68/95 (72%) dogs. Clinical hypoglycemia was observed in 6/95 (6%) dogs. These episodes were observed in 3 dogs treated with IGla300 q24h, 2 dogs treated with IGla300 q12h, and 1 dog receiving IGla300 q12h and porcine lente as bolus insulin q24h. Most episodes were mild, with 2 dogs (1 treated with IGla300 q24h and the other with IGla 300 q12h) developing severe signs that included seizures. No dogs developed DKA during the study period.

### 3.4 Dogs with concurrent diseases

One or more concurrent diseases were documented in 57/95 (60%) dogs. The most common diseases included naturally occurring hypercortisolism (20/57, 35%), chronic gastroenteropathy (10/57, 18%), and acute or chronic pancreatitis (8/57, 14%). The most common medications administered concurrently included trilostane (17/57, 30%), maropitant citrate (13/57, 23%), topical (10/57, 18%) or systemic (6/57, 11%) glucocorticoids, pancreatic enzymes (6/57, 11%), and fenofibrate (5/57, 9%).

**FIGURE 1:**
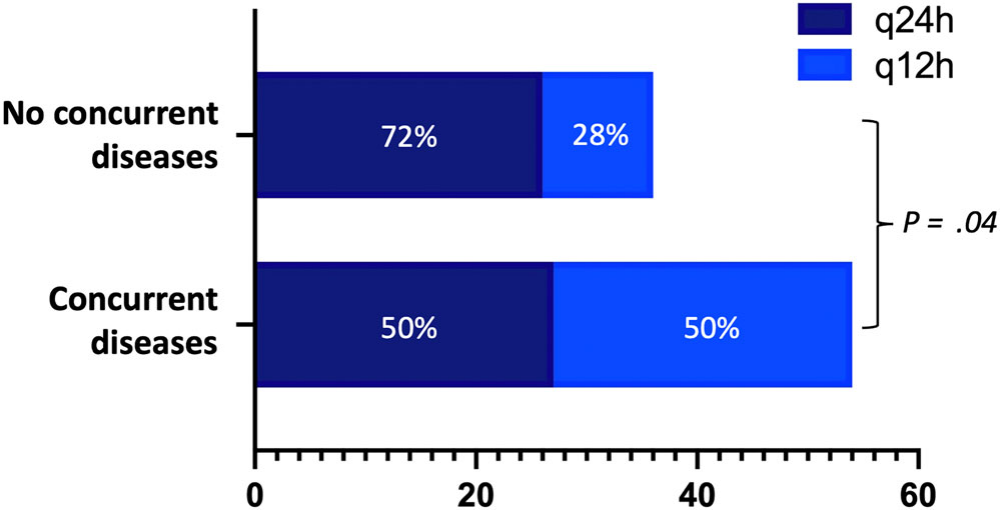
Comparison of insulin administration frequency between dogs with concurrent diseases (n = 54) and those without (n = 36). Dogs receiving meal‐time bolus injections (n = 5) were excluded from the statistical analysis. Data are presented as frequencies.

**FIGURE 2:**
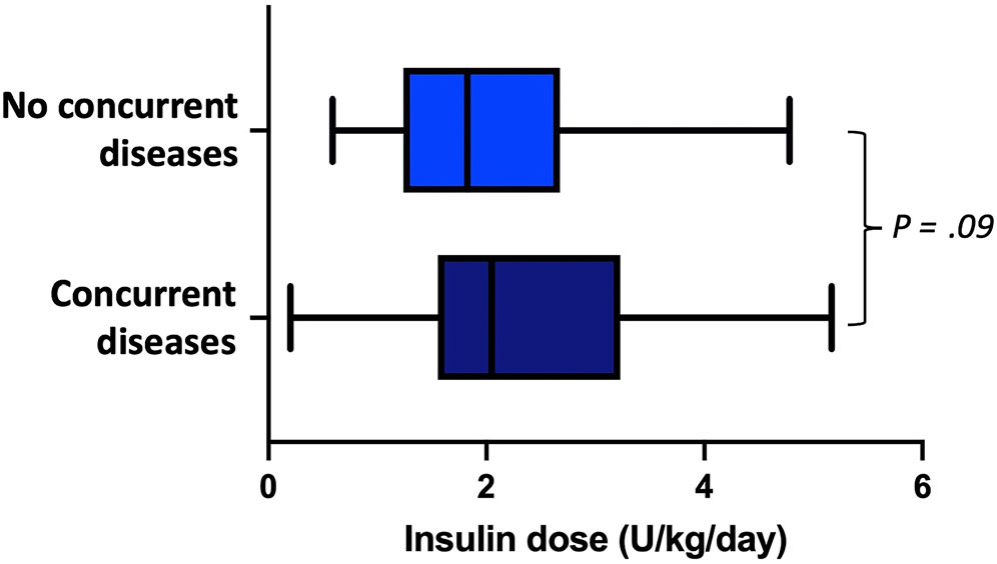
Box plots of the total insulin dose in dogs with (n = 57) or without (n = 38) concurrent diseases. The vertical bars in the middle of the box represent the median value.

At the end of the study period, 29/57 (51%) dogs with concurrent diseases were receiving IGla300 q24h, and 28/57 (49%) dogs IGla300 q12h. Meal‐time bolus injections (NPH insulin [n = 2], porcine lente insulin [n = 1]) were added in 3 dogs with concurrent diseases (2 dogs receiving IGla300 q24h and 1 dog receiving IGla300 q12h). Dogs without concurrent diseases were more likely to receive IGla300 q24h when compared with dogs with concurrent diseases (72% vs 50%, *P* = .04; Figure 1). The median total insulin dose in dogs with concurrent diseases was 2.1 (0.2‐5.2) U/kg/day. No differences were observed in the median total insulin dose between dogs with and without concurrent diseases (*P* = .09, Figure 2). In dogs with concurrent diseases, glycemic control was classified as excellent in 38/57 (67%) dogs, good in 17/57 (30%) dogs, and moderate or poor/insufficient in 1 dog each. No differences were observed in levels of glycemic control between dogs with and without concurrent diseases (*P* = .47). The median time to achieve glycemic control in dogs with concurrent diseases was 17 (4‐99) days. The number of days required to achieve glycemic control did not significantly differ between dogs with and without concurrent diseases (*P* = .81). Clinical hypoglycemia was observed in 4/57 (7%) dogs with concurrent diseases and 2/38 (5%) dogs without concurrent diseases. One of the 2 dogs that experienced severe signs of hypoglycemia had concurrent diseases.

## 4 DISCUSSION

Our study demonstrates the clinical utility of a protocol for dose titration of IGla300 in diabetic dogs using continuous glucose monitoring, providing a practical alternative to traditional treatment approaches involving 12‐hourly injections of intermediate‐acting insulin formulations combined with consistent meal feeding. The management of DM in dogs aims to resolve or improve clinical signs, minimize potential complications, and ensure a high quality of life for both the dog and the owner.^26^ Given the high risk of euthanasia for diabetic dogs if the owner feels unable to cope with the requirements of treatment, maintenance of the companion animal‐human bond should be prioritized when discussing therapeutic options.^28^ Six of the top 10 concerns reported by owners of diabetic dogs relate to the impact of the daily treatment schedule on their quality of life.^29^ Therefore, provision of the practical alternative reported here with more flexible feeding options and once or twice daily insulin dosing will likely ease the burden for many caregivers of diabetic dogs. The safe and timely dose titration protocol using FGMS also will allow clinicians to resolve or improve the clinical signs of DM within a relatively short timeframe.

In our study, administration of IGla300 was associated with good or excellent glycemic control in most dogs, including dogs with concurrent diseases. Continuous glucose monitoring facilitated clinical and glycemic improvement within a relatively short time period, and the majority of dogs (72%) were considered clinically controlled within the first month of treatment. The median time to achieving glycemic control was only 17 days, much shorter than previously reported for other insulin preparations (and different monitoring schemes) commonly used in dogs with DM.^30^, ^31^, ^32^, ^33^ In a study of 53 diabetic dogs treated with porcine lente insulin and monitored with traditional BGCs, the median duration of time to achieving dose equilibration was 35 days.^30^ Similar results were reported in 10 diabetic dogs treated with IGla100 and monitored weekly with BGCs, in which a median of 38 days was required to achieve stable insulin doses.^31^ In diabetic dogs treated with insulin detemir and NPH, glycemic control was classified as good in 30% and 73% of cases, respectively, at the end of the 6‐month (detemir) and 3‐month (NPH) study periods.^32^, ^33^ In a more recent study evaluating the efficacy of protamine zinc insulin (PZI) in 276 diabetic dogs and using BGCs, glucose parameters stabilized at day 42 after treatment initiation.^34^ We believe that the shorter time required to achieve glycemic control in our study was the result of using FGMS for monitoring in combination with the administration of an insulin formulation associated with relatively low day‐to‐day variability.^7^ Additional studies will be needed to assess the relative contributions of using IGla300 vs monitoring with FGMS to the results reported here.

Dose titration for IGla300 is feasible only if patients are monitored using the FGMS, because decisions on the frequency of insulin administration and the introduction of bolus insulin can only be made using day‐to‐day and 24‐hourly assessments of IG concentrations. The guidelines we used for dose adjustments in our study contrast with those reported for other insulin formulations.^35^, ^36^ Traditional recommendations were that insulin doses should not be adjusted more frequently than every 7 to 14 days.^35^, ^36^ In our study, dose adjustments were recommended every 1 to 3 days, likely contributing to achieving glycemic control in a shorter timeframe. Rapid dose adjustments were deemed safe because of the inherently low day‐to‐day variability of IGla300, and were facilitated by the relatively intensive monitoring provided by the FGMS.^7^ To the best of our knowledge, ours is the first study in which insulin dose titration in client‐owned diabetic dogs relied on FGMS monitoring in the home environment. Previously, serial BGCs have been the most commonly recommended method for guiding insulin dose adjustments in diabetic dogs,^35^, ^36^ These have several disadvantages, such as the need for repeated blood sampling and the risk of missing the blood glucose peak or nadir.^20^ Importantly, BGCs do not allow easy assessment of glycemia on consecutive days. This concern limits their usefulness, especially for assessment of older insulin formulations that are associated with substantial day‐to‐day variability,^7^ necessitating a more cautious approach to dose titration. In recent years, glucose monitoring has been revolutionized by the use of the FGMS.^37^ The FGMS allows real‐time and comprehensive assessment of glycemic excursions occurring throughout the day and night, as well as of glucose variations over consecutive days, enabling clinicians to make quicker and more informed decisions about insulin dose titration.^37^ However, monitoring using FGMS is acknowledged to be relatively intensive and more costly than some simpler monitoring strategies. Consequently, some diabetic pet owners might not want to continue using the FGMS long‐term.^38^ After completing the dose titration period for IGla300, less intensive monitoring options often will be appropriate for ongoing long‐term management of the dog's DM.

In dogs, IGla300 is reported to have lower potency compared with other insulin preparations,^17^ which typically results in a lower risk of hypoglycemia and may require higher therapeutic doses. It also might provide advantages, especially in small dogs, allowing small adjustments in the effective dose with every unit change. The median dose of IGla300 to achieve glycemic control was 1.9 U/kg/day (with a range up to 5.2 U/kg/day), which is higher than doses typically required to achieve glycemic control in dogs treated with other insulin formulations.^30^, ^31^, ^32^, ^33^, ^34^ However, the effective dose range in our study might have been over‐estimated because of the presence of concurrent diseases in 60% of the dogs. Although we initiated our protocol with a low starting dose of 0.5 U/kg q24h, rapid dose titration facilitated by use of the FGMS was crucial to treatment success because it minimized the period of under‐dosing in dogs that eventually required higher doses.

The option of q24h insulin administration provides substantial practical benefit for the owners of diabetic dogs because it essentially halves the potential impact on their lifestyle compared with a q12h schedule. In our study, glycemic control was achieved with q24h administration of IGla300 in 50% and 72% of the dogs with and without concurrent diseases, respectively. Insulin formulations that are most commonly used in dogs, such as porcine lente, NPH, and detemir, typically are administered q12h.^36^ Recently, q24h administration was reported in 135 of 224 otherwise healthy diabetic dogs treated with PZI.^34^ In that study however, only 57% of dogs were still on q24h dosing at the end of the study period,^34^ compared with 72% of dogs without concurrent diseases in our study. Importantly, clinical hypoglycemia was reported in 9% and seizures in 6% of the dogs in the PZI study. It is unclear how many of these hypoglycemic events were specifically associated with q24h administration of PZI, but it is likely that the risk of hypoglycemia increases with lower frequency of administration of PZI. In comparison, only 5% of dogs without concurrent diseases in our study experienced hypoglycemia, with seizures occurring in only 1 dog. However, comparison between the 2 studies is challenging because of differences in monitoring protocols and study populations, including differences in the proportion of insulin‐naive dogs (15% IGla300 vs 56% PZI) and the exclusion of dogs with concurrent disease from the PZI study (in contrast to 60% of dogs with concurrent diseases in our study). Of note, the frequency of hypoglycemia observed in our study also was much lower than that reported in dogs treated with q12h porcine lente (38.6%), insulin detemir (40%), and IGla100 (20%).^30^, ^31^, ^32^, ^33^ The lower rate of clinical hypoglycemia in our study could be explained by the low potency and low day‐to‐day variability of IGla300 and the meticulous monitoring facilitated by use of the FGMS.

The use of a basal insulin in dogs represents a paradigm shift in the overall strategy of DM treatment in dogs, because it uncouples insulin injections from feeding and provides owners with more flexibility in terms of meal timing, type of food, and consistency. In our study, the feeding method frequently was changed from a strict q12h feeding at the times of insulin injections to more frequent feeding of smaller meals or a different twice daily feeding schedule. This approach is in contrast to the traditional rigid recommendation of dividing the daily caloric intake into 2 meals that must be fed (and then consumed in full and digested) at the times of insulin injections in order to avoid hypoglycemia.^33^, ^34^ Critically, the use of a basal insulin allows for meals to be skipped without risking hypoglycemia. This approach is translated into decreased stress for owners and better control on days when meals are either deliberately withheld (eg, in preparation for anesthesia), refused, or vomited.

The addition of bolus insulin was deemed necessary in only 5% of dogs to control substantial periods of high IG after ≥1 meals per day. This result was not surprising, considering our previous experience with a once‐weekly basal insulin that was associated with good glycemic control in dogs without the addition of bolus insulin.^39^ Importantly, a critical difference between DM management in dogs and people lies in treatment goals. In dogs, the main goal is clinical control, whereas in people, euglycemia is desired. Therefore, in dogs, postprandial hyperglycemia does not necessarily require treatment, unless its magnitude leads to clinical signs. If choosing to add bolus insulin, the time‐action profile of the exogenous bolus insulin should mimic physiologic bolus insulin secretion in the healthy animal. Considering physiologic bolus insulin secretion in dogs,^40^, ^41^, ^42^ intermediate‐acting insulin might provide a better approximation of bolus insulin secretion in many dogs than rapid‐acting formulations that typically are used for this purpose in diabetic people.^2^ In our study, when bolus insulin was required to manage postprandial hyperglycemia, we opted to add NPH insulin (either as NPH or as a 70/30 NPH/regular insulin mix) or porcine lente insulin once or twice daily to the treatment protocol. A starting dose of 0.25 U/kg at the time of feeding was used, and it should be noted that the final recommended doses all were higher (median, 0.5 U/kg).

Our study had several limitations, including the subjective assessment of glycemic control. We chose not to categorize glycemic control based on FGMS data because the FGMS data was used for making treatment decisions. For glycemic control categorization, a 4‐point scoring system was employed instead of the ALIVE diabetic clinical score.^26^ The latter, which is excellent for standardization in retrospective studies, is not useful for decision‐making because it lacks a threshold for treatment. Additionally, a dog might have a low score but still require a dose increase (eg, 3/12 score in a dog with severe polyuria and polydipsia). The 4‐point scoring system enabled us to summarize the clinical assessment into a single number and allowed clinicians to assess clinical signs as they deemed appropriate. Another potential limitation is the enrollment of dogs previously treated with insulin and dogs with concurrent diseases. In this population, the positive clinical outcomes of IGla300 and the monitoring protocol we used might therefore be underestimated. The decision to include dogs already on treatment and with concurrent diseases was made in order to evaluate IGla300 in a heterogenous population that was as similar as possible to that encountered in a clinical setting, at least that of referral practices. The potential underestimation of treatment success only emphasizes the potential utility of the protocol described in our study. The use of a control group would have been advantageous as a direct comparison between IGla300 with more commonly used insulin products. However, doing so would have required resources beyond those available to us at the inception of the study. Moreover, any insulin formulation chosen as control would have represented a single option out of a large variety of insulin formulations available. Finally, the fact that all treatment and monitoring expenses were borne by the owners might have biased the study toward perceived success of the treatment.

In conclusion, basal insulin treatment of diabetic dogs with IGla300 provides a practical alternative to traditional treatment approaches using q12h injections of intermediate‐acting insulin formulations and regular feeding of meals. This novel protocol represents a paradigm shift in the overall strategy of DM treatment in dogs, because it uncouples insulin injections from feeding, providing owners with more flexibility in terms of timing, type, and consistency of meals. It thus provides an opportunity to improve the quality of life and alleviate the treatment burden for many caregivers of diabetic dogs. Administration of IGla300 was associated with good or excellent glycemic control and low frequency of clinical hypoglycemia in most dogs, including dogs with concurrent diseases. Once‐daily administration of IGla300 achieved good glycemic control in the majority of diabetic dogs without concurrent diseases and in half of dogs with concurrent diseases. In some dogs, q12h administration is required and, in a few, the addition of meal‐time bolus insulin might be necessary. Dose titration for IGla300 is only feasible if patients are initially monitored using FGMS. After completing the dose titration period, less intensive monitoring methods can be employed.

## CONFLICT OF INTEREST DECLARATION

Authors declare no conflict of interest.

## OFF‐LABEL ANTIMICROBIAL DECLARATION

Authors declare no off‐label use of antimicrobials.

## INSTITUTIONAL ANIMAL CARE AND USE COMMITTEE (IACUC) OR OTHER APPROVAL DECLARATION

Approved by the Scientific Ethics Committee of the University of Bologna (protocol number 101123/2023) and by the University of Florida IACUC (protocol number 202300000146).

## HUMAN ETHICS APPROVAL DECLARATION

Authors declare human ethics approval was not needed for this study.
